# Identification of novel testing matrices for African swine fever surveillance

**DOI:** 10.1177/1040638720954888

**Published:** 2020-09-23

**Authors:** John Flannery, Martin Ashby, Rebecca Moore, Sian Wells, Paulina Rajko-Nenow, Christopher L. Netherton, Carrie Batten

**Affiliations:** The Pirbright Institute, Pirbright, Woking, Surrey, UK

**Keywords:** African swine fever virus, LAMP, pigs, real-time PCR, surveillance

## Abstract

African swine fever (ASF) is a devastating viral disease of pigs and wild boar, and it threatens global food security. We aimed to identify suitable sample matrices for use in ASF surveillance programs. Six pigs inoculated with ASFV were sampled at postmortem. Blood, bone marrow, ear biopsies, and oral, nasal, and rectal swabs were taken from all pigs. All samples were analyzed using 3 real-time PCR (rtPCR) assays and a LAMP assay. ASFV was detected at > 10^7^ genome copies/mL in blood; bone marrow was found to provide the highest viral load. Ct values provided by the rtPCR assays were correlated, and ASFV was detected in all oral, nasal, and rectal swabs and in all ear biopsy samples irrespective of the location from which they were taken. The LAMP assay had lower sensitivity, and detected ASFV in 54 of 66 positive samples, but delivered positive results within 17 min. We identified additional sample matrices that can be considered depending on the sampling situation: bone marrow had a high probability of detection, which could be useful for decomposed carcasses. However, ear biopsies provide an appropriate, high-throughput sample matrix to detect ASFV and may be useful during surveillance programs.

African swine fever (ASF) is a highly pathogenic viral disease of pigs and wild boar caused by the ASF virus (ASFV; *Asfarviridae*, *Asfivirus*).^2^ The unprecedented Eurasian spread of ASFV since 2007 is of considerable concern to veterinary authorities and threatens global food security. In the absence of an approved vaccine, control of ASF is reliant on international cooperation, enhanced biosecurity, and appropriate surveillance strategies. In the United Kingdom, and in line with European Union animal health regulations, ASF diagnosis is confirmed using 2 independent antigen-detection methods (antigen ELISA and a PCR assay, or 2 PCR assays targeting different regions of the genome).^2^ Real-time PCR (rtPCR) is the most suitable detection method, and prescribed assays exist.^3,7^ Alternative test methods, such as lateral flow devices (LFDs) and loop-mediated isothermal amplification (LAMP), can provide rapid and less expensive field-based detection and potentially expedite control measures.^6,9^

ASFV causes high viremia, thus EDTA blood and tissues such as lymph nodes, spleen, lung, and kidney are chosen as the most appropriate sample matrices for testing. In autolyzed carcasses, these tissues may be unsuitable, thus bone marrow is considered a suitable matrix.^2^ The use of swabs has been investigated^5^; however, EDTA blood was found to be the most reliable detection matrix.

Active surveillance, which could entail obtaining blood samples from thousands of pigs, would present a significant undertaking. Biopsies can be obtained easily from the ear, causing minimal discomfort to the animal and can overcome some of the difficulties in blood sampling, particularly in the field. Passive surveillance that relies on obtaining swine carcasses can present sampling considerations given that there may be few acceptable matrices available following decomposition. Thus, we investigated the suitability of swabs and ear biopsies for use during active surveillance and the use of bone marrow during passive surveillance regimes. Expanding the suite of suitable sample matrices will be of interest to veterinary policy leads in countries at risk of or controlling ASF.

Animal experiments were carried out under the Home Office Animals (Scientific Procedures) Act 1986 (https://www.legislation.gov.uk/ukpga/1986/14/contents) and were approved by the Animal Welfare and Ethical Review Board of The Pirbright Institute. The animals were housed in accordance with the Code of Practice for the Housing and Care of Animals Bred, Supplied or Used for Scientific Purposes (https://assets.publishing.service.gov.uk/government/uploads/system/uploads/attachment_data/file/388535/CoPanimalsWeb.pdf), and bedding and species-specific enrichment were provided throughout the study to ensure high standards of welfare. Through careful monitoring, pigs that reached the scientific or humane endpoints of the study were euthanized by an overdose of anesthetic. All procedures were conducted by Personal License holders who were trained and competent and under the auspices of Project Licenses.

Three female, 15-wk-old Large White Landrace Hampshire pigs were infected via the intramuscular route in the rump with a genotype I (GI) ASFV (ASFV OUR T88/1), and 3 were infected with a genotype II (GII) ASFV (ASFV Georgia 2007/1). Pig 1 was euthanized 4 d post-infection (dpi) and the other 5 animals at 5 dpi. A sample panel comprising EDTA blood, swabs (oral, nasal, and rectal), and bone marrow collected from the humerus and rib were taken from each animal. In addition, 2.5-mm biopsy punches (Integra Miltex; Biosave) were obtained from 6 locations on the left ear of each animal. Oral, nasal, and rectal swabs, as well as biopsy punches, were taken after euthanasia, but before the animals were exsanguinated.

Bone marrow and ear biopsy samples were added to 2-mL lysis tubes (Lysing matrix D; MPBio) containing 1 mL of PBS. Homogenization was performed using a microtube homogenizer (BeadBug; MilliporeSigma) set at 3,000 rpm for 360 s. The homogenate was centrifuged at 3,000 × *g* for 5 min and the supernatant transferred to a new 2-mL tube. The swabs were placed in 1 mL of PBS and allowed to stand for 60 s before the eluate was transferred to a new 2-mL tube.

DNA was extracted in duplicate using 100 µL of homogenate, EDTA blood, or swab eluate, and automated extraction was performed (KingFisher Flex, LSI MagVet extraction kit; Thermo Fisher). ASFV positive and negative controls were included in the extraction, and DNA was eluted into 80 µL of elution buffer. DNA was stored at 4°C prior to analysis.

Real-time PCR was performed in duplicate (Applied Biosystems 7500 fast instrument, VetMAX ASFV qPCR kit; Thermo Fisher) using the TaqMan^7^ and Universal Probe Library (UPL)^3^ assays that each target ASFV VP72. The ASFV LAMP was performed using the assay on a portable fluorimeter (GenieII; OptiGene).^3,6,7^

At postmortem, there was no significant difference between the viral load in GI- or GII-infected groups (*p* = 0.25). Mean ASFV concentrations were 8.8 and 8.4 log_10_ genome copies/mL EDTA blood for GI- and GII-infected animals, respectively. ASFV was detected in all sample matrices (Table 1). All swabs taken (*n* = 36) were positive for ASFV, and cycle threshold (Ct) values were 23.4–36.3 for oral swabs, 23.9–33.1 for nasal swabs, and 29.6–39.5 for rectal swabs; no significant difference existed between sample matrices (one-way ANOVA, *p* = 0.084). Bone marrow Ct values were 18.2–23.8, and no significant difference was found between humerus and rib Ct values (*t* test, *p* = 0.576).

**Table 1.** Mean TaqMan^7^ real-time PCR Ct values for African swine fever virus obtained from alternative testing sample matrices.

Matrix	TaqMan mean Ct value						Mean Ct value
	Genotype I			Genotype II			
	Pig 1, 5 dpi	Pig 2, 5 dpi	Pig 3, 4 dpi	Pig 4, 5 dpi	Pig 5, 5 dpi	Pig 6, 5 dpi	
Swab							
Oral	27.9	32.4	23.4	36.3	29.1	25.6	29.1
Nasal	27.9	29.4	23.9	33.1	32.5	30.3	29.5
Rectal	31.3	32.7	29.6	35.8	35.2	39.5	34.0
Bone marrow							
Humerus	18.2	23.8	19.3	21.9	20.2	19.0	20.4
Rib	17.1	19.1	21.7	20.0	19.1	21.5	19.7
Ear biopsy							
1	29.4	31.8	28.6	31.2	26.4	25.9	28.9
2	28.7	29.7	28.6	27.5	24.8	27.1	27.7
3	28.8	30.7	27.0	28.6	27.5	29.3	28.6
4	29.5	29.6	29.3	30.7	27.9	27.8	29.1
5	27.0	28.7	28.8	30.3	28.2	29.2	28.7
6	29.7	29.5	27.9	29.2	28.2	30.6	29.2

Ct = cycle threshold.

In all ear biopsy samples, ASFV was detected with Ct values of 24.8–31.8 irrespective of the location from which it was taken (Fig. 1), supported by a one-way ANOVA (*p* = 0.617). The UPL and VetMAX rtPCR assays showed complete agreement with the TaqMan^7^ assay (Suppl. Table 1), and Ct values were correlated (*r* > 0.963). The LAMP assay indicated a positive result in 54 of 66 samples, with a mean time to positivity (*t_p_*) of 11.08 min (range 06:57–17:75 min) and had a detection limit equivalent to a Ct value of 30–32. In samples with a Ct < 30, the LAMP provided equivalent positive results to rtPCR, and a correlation between Ct value and *t_p_* was found (Pearson correlation *r* = 0.676, *p* < 0.001).

**Figure 1.**
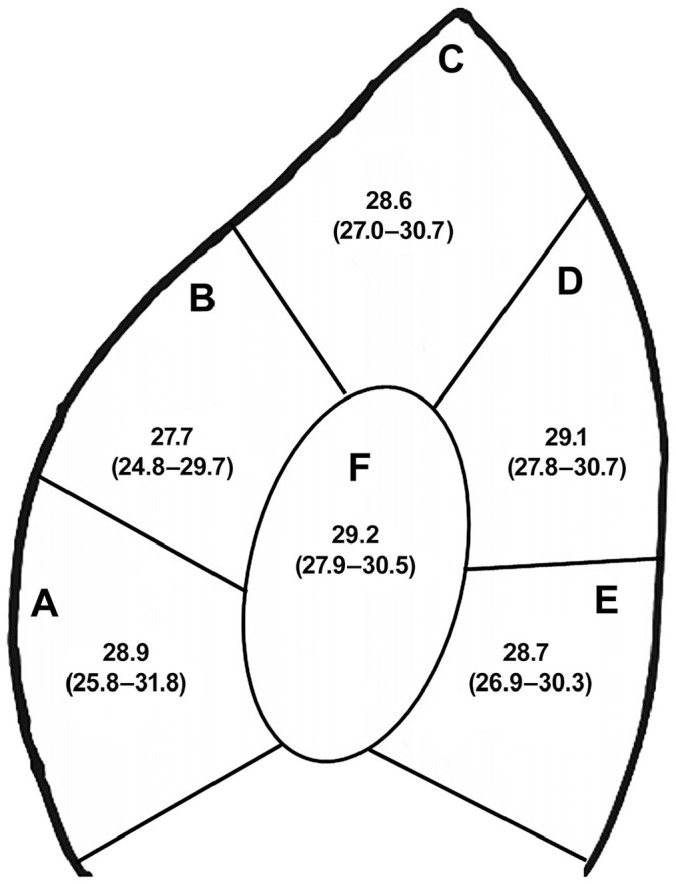
Mean cycle threshold (Ct) values obtained from 6 pig ear biopsy sites. Shown is the area of the ear that was sampled (**A**–**F**), the mean Ct value, and the range of Ct values (in parentheses) obtained from 6 animals using the TaqMan^7^ real-time PCR assay.

Our findings support the validity of these samples for detection of ASFV if taken from a deceased animal, decomposed carcass, or a symptomatic animal in the field. We performed analysis using front-line ISO/IEC-accredited rtPCR assays and a non-accredited LAMP assay. The preliminary identification of ASFV-positive samples in the field can expedite control measures until laboratory confirmation has been provided. Our results are in agreement with other studies on the use of swabs to detect ASFV, although the detection can be intermittent.^4,5^ Bone marrow yielded the highest concentration of ASFV, which again supports its use for sampling autolyzed or decomposed carcasses, as has been shown in the Baltic countries and more recently in Belgium.^1,8^ An important finding of our work is that we have identified the ear as a suitable matrix to detect ASFV in infected pigs. Given the difficulties of sampling pigs, particularly en masse during surveillance or outbreak situations, an ear biopsy (punch) can provide a much quicker alternative sampling matrix than EDTA blood and should allow for higher throughput sampling in the field. Given the threat of ASFV incursion, increasing the suite of suitable testing sample matrices will expedite detection and support potential surveillance programs.

## Supplemental Material

Supplemental_material – Supplemental material for Identification of novel testing matrices for African swine fever surveillance Supplemental material, Supplemental_material for Identification of novel testing matrices for African swine fever surveillance by John Flannery, Martin Ashby, Rebecca Moore, Sian Wells, Paulina Rajko-Nenow, Christopher L. Netherton and Carrie Batten in Journal of Veterinary Diagnostic Investigation
