# Supplementary material for: Identification of novel testing matrices for African swine fever surveillance
Source: J Vet Diagn Invest. 2020 Sep 23;32(6):961–3. doi: 10.1177/1040638720954888 (PMC7645617; doi:10.1177/1040638720954888)
Supplement: Supplemental_material – Supplemental material for Identification of novel testing matrices for African swine fever surveillance [file Supplemental4_material.pdf]

Flannery J, et al. Identification of novel testing matrices for African swine fever surveillance

Supplementary Table 1. Mean Ct values achieved by all rtPCR assays and the LAMP assay.

	rtPCR assay mean Ct value			LAMP
Animal/Sample matrix	King	UPL	VetMAX	t_p
Pig 1				
Fecal swab	31.3	30.2	29.9	10:57
Nasal swab	27.9	26.5	26.1	09:12
Oral swab	27.9	27.4	27.0	08:57
Bone marrow, humerus	18.2	17.8	17.2	06:57
Bone marrow, rib	17.1	17.0	16.9	07:57
Ear biopsy 1	29.4	29.0	28.5	10:27
Ear biopsy 2	28.7	28.1	27.8	10:27
Ear biopsy 3	28.8	28.4	27.9	09:57
Ear biopsy 4	29.5	29.6	29.1	10:27
Ear biopsy 5	26.9	26.5	26.3	09:27
Ear biopsy 6	29.7	28.8	28.7	10:57
Pig 2				
Fecal swab	32.7	31.3	31.0	12:45
Nasal swab	29.4	28.4	27.8	11:45
Oral swab	32.4	32.2	31.4	14:00
Bone marrow, humerus	23.8	23.7	23.3	08:15
Bone marrow, rib	19.1	18.6	18.9	07:30
Ear biopsy 1	31.8	30.8	30.3	12:00
Ear biopsy 2	29.7	29.4	29.3	10:30
Ear biopsy 3	30.7	30.4	30.0	11:30
Ear biopsy 4	29.6	29.7	29.3	10:30
Ear biopsy 5	28.7	28.5	28.3	10:30
Ear biopsy 6	29.5	29.1	28.8	12:00
Pig 3				
Fecal swab	29.6	32.3	31.0	14:45
Nasal swab	23.9	26.5	25.2	11:00
Oral swab	23.4	27.1	25.3	11:00
Bone marrow, humerus	19.3	22.2	20.4	08:30

Bone marrow, rib	21.7	20.8	19.4	08:45
Ear biopsy 1	28.6	31.1	29.1	15:00
Ear biopsy 2	28.6	30.6	29.3	13:15
Ear biopsy 3	27.0	29.5	28.2	13:00
Ear biopsy 4	29.3	31.8	30.3	12:15
Ear biopsy 5	28.8	30.8	29.2	12:00
Ear biopsy 6	27.9	30.2	28.9	12:00
Pig 4				
Fecal swab	35.8	37.0	38.0	-
Nasal swab	33.1	32.9	31.9	15:00
Oral swab	36.3	35.0	35.4	-
Bone marrow, humerus	21.87	21.68	21.52	08:15
Bone marrow, rib	19.99	19.63	19.59	08:00
Ear biopsy 1	31.20	30.94	30.12	12:15
Ear biopsy 2	27.45	27.41	27.13	10:00
Ear biopsy 3	28.61	28.29	27.83	12:15
Ear biopsy 4	30.74	30.43	29.60	14:45
Ear biopsy 5	30.27	29.92	29.33	12:30
Ear biopsy 6	29.19	29.09	28.38	11:45
Pig 5				
Fecal swab	35.19	35.01	34.04	-
Nasal swab	32.49	32.95	31.94	-
Oral swab	29.14	30.41	29.40	14:45
Bone marrow, humerus	20.22	20.75	19.97	09:00
Bone marrow, rib	19.07	19.56	18.77	09:00
Ear biopsy 1	26.38	27.45	26.19	13:00
Ear biopsy 2	24.81	25.75	24.66	12:00
Ear biopsy 3	27.45	28.32	27.18	12:00
Ear biopsy 4	27.90	28.65	27.94	11:45
Ear biopsy 5	28.16	28.53	27.87	13:00
Ear biopsy 6	28.16	28.53	27.70	13:15
Pig 6				
Fecal swab	39.49	36.48	35.67	-
Nasal swab	30.34	31.63	30.62	18:15
Oral swab	25.65	26.76	26.11	08:30
Bone marrow, humerus	19.00	19.56	19.24	09:00
Bone marrow, rib	21.53	21.84	21.50	08:45
Ear biopsy 1	25.82	27.13	26.30	12:30

Ear biopsy 2	27.07	27.75	26.90	14:30
Ear biopsy 3	29.32	30.55	29.37	-
Ear biopsy 4	27.78	28.91	28.14	12:00
Ear biopsy 5	29.19	30.14	29.32	17:45
Ear biopsy 6	30.55	31.48	30.53	16:00

Ct = cycle threshold; LAMP = loop-mediated isothermal amplification; rtPCR = real-time PCR; t_p = time to positivity; UPL = Universal Probe Library; VetMAX = VetMAX ASFV qPCR kit; Thermo Fisher.
